# Building the gateway to consciousness—about the development of the thalamus

**DOI:** 10.3389/fnins.2013.00094

**Published:** 2013-06-04

**Authors:** Steffen Scholpp, Tomomi Shimogori

**Affiliations:** ^1^Institute of Toxicology and Genetics, Karlsruhe Institute of TechnologyKarlsruhe, Germany; ^2^Brain Science Institute RIKEN, Lab for Molecular Mechanisms of Thalamus DevelopmentSaitama, Japan

The thalamus is a twinned bulb-shaped structures that form at the top of the brainstem on either side of the third ventricle. The thalamic complex is located in the posterior forebrain and includes the prethalamus and thalamus (formerly known as ventral thalamus and dorsal thalamus, respectively). This complex is the major sensory relay station of the brain, receiving all inputs (except olfaction) and connecting reciprocally with the overlying cortex therefore, Crick and Koch (2003) has described the thalamus as “the gateway to consciousness.” Although, the thalamus has been anatomically characterized in vertebrates the underlying genetic mechanism leading to the formation of this complex brain area is largely unknown.

**Figure 1:**
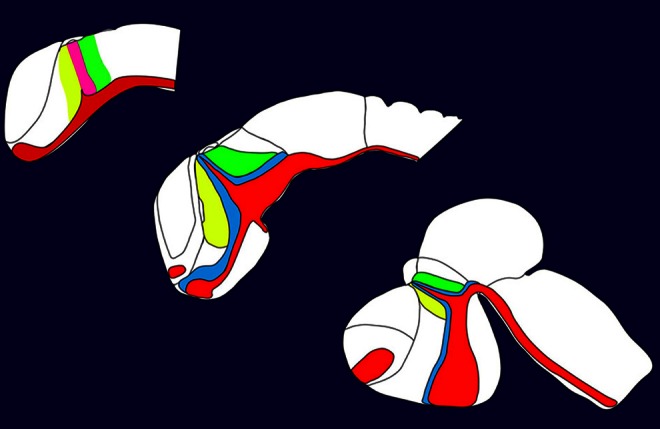
**Development of the thalamus in vertebrates**.

In this special issue we tried to cover many aspects describing the development of the posterior forebrain i.e., the thalamus. The posterior forebrain can be subdivided in the epithalamus, the prethalamus (Figure 1, indicated in yellow), the thalamus (Figure 1, indicated in green), and the pretectum. Mechanisms regulating the formation and setting the boundaries between them are described in the article of Chatterjee and Li (2012). The crucial structure for the development of the thalamus is a small group of cells secreting different signaling molecules and is localized at the intrathalamic boundary, the zona limitans intrathalamica (ZLI). This cell population has been termed the mid-diencephalic organizer (MDO) or alternatively as ZLI organizer. The MDO releases three different families of signaling factors, Shh, Wnt, and Fgf. This network has been described in the articles of Hagemann and Scholpp (2012) focusing mainly on events in zebrafish and in Martinez-Ferre and Martinez (2012), who describe the situation in chick. Both group described that the principal signal of the MDO is Shh, which is conserved in different animals (Figure 1, expression domain of Shh indicated in red). Therefore, we elucidate the function of Shh in mice more in detail in two articles. Epstein (2012) focuses in his article on the description of the function of this signaling factor, whereas Haddad-Tóvolli et al. (2012) focuses on the regulators downstream of Shh signaling, the GLI transcription factors. Pre-patterning of the thalamic tissue is essential for the axonal projections of the thalamus and the most important axonal output of this structure is the thalamo-cortical projection. Price et al. (2012) and Grant et al. (2012) review recent progress of the development of these projections and axonal guidance. The zebrafish serves as a novel vertebrate model organism in thalamic development, however, the comparability to the mammalian systems is difficult. In a comparative review (Mueller, 2012) summarizes anatomical similarities and differences of the thalamus in different model organisms. Dorsally adjacent to the thalamus is the location of the habenula. The development of the connection to the thalamic tissue has been elucidated by two articles from Beretta et al. (2012) and Aizawa et al. (2011).

After our opinion, the special issue “Building the gateway to consciousness—about the development of the thalamus” summarizes recent efforts in understanding the formation of thalamus in vertebrates and we carefully selected carefully articles covering the entire field. However, it is impossible to elucidate any aspect in sufficient detail and we would like to apologize to our colleagues for potential gaps. We wish all scientist as much as fun in reading the articles as we had in assembling it and sincerely hope that this special issue helps the field of thalamus development to find the scientific recognition it deserves.
